# Report of the Distribution Committee

**Published:** 1919-12-20

**Authors:** 


**King Edward's Hospital Fund—(*continued*).**

### REPORT OF THE DISTRIBUTION COMMITTEE.

1. The President and General Council have this year appropriated the sum of £218,000 for distribution amongst the London hospitals, as against £190,000 in 1918, an increase of £28,000.

2. This increase was authorised partly in consequence of a report of the Distribution Committee drawing attention to the exceptional expenditure which hospitals would have to incur on renewals and repairs deferred during the war. The committee have accordingly dealt with the greater part of the additional £28,000, not as a permanent increase in the annual distribution, but as a special addition to this year's total to assist the hospitals in meeting an exceptional liability which arises directly out of the war. As, however, renewals and repairs, even if exceptional in amount in any particular year, are none the less part of the ordinary cost of maintaining a hospital, the assistance afforded by the Fund for this purpose is given as part of its ordinary contribution to maintenance; and the only visible result is that the grants to maintenance are larger than they would have been had the financial considerations been based, in the usual way, solely on last year's audited accounts.

3. The number of hospitals applying for grants is 107.

4. Of the total amount distributed, £174,625 has been applied in the form of grants to maintenance. This is an increase of £14,375 over the corresponding total in 1918.

5. As was to be expected, an exceptionally large number of applications was received for grants in aid of improvement or extension, since the hospitals are now proceeding with the preparation of plans which had been deferred during the war. The capital grants made by the Fund this year amount to £43,375, as compared with £29,750 in 1918, an increase of £13,625. This year's total includes £12,025 in reduction of capital liabilities already incurred, the remaining £31,350 being earmarked for schemes which it is proposed to carry out in the more or less near future.

6. The schemes to which this sum of £31,350 is contributed this year do not by any means represent the whole of the applications for capital grants. For various reasons the committee thought it best to postpone the consideration of several of the more important applications till the next distribution. In the first place, some of the schemes are not yet sufficiently mature. In the second place, many of them involve the provision of additional beds, the full consideration of which has been deferred, for the reason given in a later paragraph, till such time as the committee could make a general survey of all such schemes instead of dealing with them one by one. In the third place, the Fund will, in the early part of 1920, have an opportunity of making a survey of a very large number of schemes which are being and will be submitted in connection with the forthcoming distribution of surplus funds entrusted to the King's Fund by the Joint Committee of the British Red Cross Society and the Order of St. John of Jerusalem in aid of improvements or extensions to hospitals in the London area which can and will treat ex-soldiers or ex-sailors; and, this survey having been made, the Fund will have more complete material for considering these applications at the following ordinary distribution of the Fund in November 1920. In the fourth place, in no instance does it appear that the action of the committee in postponing the question of a grant till next autumn will delay the date at which the hospital has any prospect of beginning to build.

For these reasons the Distribution Committee have confined their capital grants this year to schemes which seem to them to be specially urgent, and schemes which have already been postponed on account of the war for a long time since they were first submitted to the Fund.

7. In their last report the committee referred to the large number of schemes of extension involving additional beds, and stated that they proposed specially to invite all the hospitals concerned to furnish them with the latest particulars of all such proposals. The details received down to the end of November show a large increase on the number reported by the committee last year. Out of 109 hospitals included in the prefatory note to the Fund's Statistical Report for 1918, more than 70 have returned particulars of additions or proposed additions to their 1913 bed accommodation, and of these 59 have schemes involving capital expenditure. There were, in 1913, 10,735 beds available in these 109 hospitals and in their country branches, and the number of additional beds for civilians (including ex-Service men) reported by those hospitals which are able to give

**King Edward's Hospital Fund—(*continued*).**

figures may be summarised as follows: Additional beds for civilians opened since 1913, after deducting beds closed, 731; additional beds provided for. either before or after 1913, but not yet opened, 331; additional beds definitely contemplated, 2,927; additional beds indefinite or future, 1,144. Besides these there would be 386 beds required to complete the original plans of hospitals which are only partially built, but the authorities of which do not at present contemplate taking any action.

8. If all these schemes were to be carried out, very formidable liabilities would be incurred both for capital cost and for annual maintenance. No detailed estimate can be given, but at the lowest probable figure the capital cost could not be less than £3,000,000 and the additional annual upkeep would exceed half a million. This capital expenditure, moreover, would be in addition to the cost of numerous schemes for bringing existing hospital accommodation up to date by the provision of new nurses' quarters, the enlargement of out-patients' departments, and the making of other much-needed improvements which do not involve more beds. It is only in exceptional cases, therefore, that the Fund can take the responsibility of encouraging any scheme until a general survey has been made of all the proposals, from the point of view of geographical distribution, comparative urgency, and financial feasibility.

The Committee have accordingly refrained from making grants in aid of bed extensions except in a few particularly urgent cases, and have notified all hospitals which contemplate applying for grants from the special distribution of the Red Cross surplus funds in aid of schemes of extension or improvement that, where additional beds are proposed, the Fund will require definite and detailed replies to its usual questions as to "the reasons and considerations which have led to the formulation of the proposals" and "the prospect of provision for future maintenance."

9. The following grants in aid of schemes of building expenditure are recommended for renewal under the two years rule:

General Lying-in Hospital, £1,000 in 1917 to improvements.

Hospital and Home for Sick Children, Sydenham (now South-Eastern Hospital for Children), £75 in 1915 to central heating.

Queen Charlotte's Lying-in Hospital, £1,000 in 1913 and £650 in 1917 to new out-patient department.

Queen's Hospital for Children, £288 12s. 11d., balance of £1,000 in 1917 to improvements in the out-patient department.

St. Mary's Hospital for Women and Children, Plaistow, £1,500 in 1913 and £1,000 in 1917 to new nurses' home and out-patient department.

Victoria Hospital, £200 in 1916 to new heating apparatus.

10. In dealing with questions of cost per bed the Committee have, as usual, distinguished between increases which are peculiar to individual hospitals and those which are due to the rise in prices and in wages, the difficulty of obtaining skilled service, the development of expensive special treatments, and other general causes. They have regarded the latter, in the absence of definite evidence to the contrary, as being covered by the average increases in cost, and have only taken into account cases where the cost is high in comparison with other hospitals or where there has been a disproportionate rise, and where the explanations tendered by the hospital concerned have seemed to them inadequate to account for the difference.

11. The Committee desire once more to express their warm thanks to the visitors, not only for the general evidence which their reports supply as to the efficiency of the various hospitals applying for grants, but also for numerous comments and suggestions, many of which the Committee recommend should be sent on with the grants to the institutions concerned. The Committee wish it to be understood that these extracts are sent in the first instance merely with a request for the observations of the Hospital Committee. The replies are communicated to next year's visitors, so that ample opportunity is given for discussion before any criticism made by this year's visitors comes up for further consideration at the next distribution.

12. At their first meeting this year the Distribution Committee passed a resolution expressing to Sir William Church their great regret at his retirement from the committee of which he had been Chairman for the past sixteen years, and placing on record their appreciation of his long and zealous service to the Fund and of the ability and courtesy with which he had presided over their meetings. They trust that he may long retain his active connection with the Fund as a member of the General Council.

13. The details of the awards to the various hospitals are appended.

For the Committee, John Tweedy, Chairman.

December 2, 1919.

